# Trends in incidence rates of acute myocardial infarction and stroke among immigrant groups in Norway, 1999–2019: the NCDNOR project

**DOI:** 10.1136/openhrt-2024-003114

**Published:** 2025-04-04

**Authors:** Kjersti Stormark Rabanal, Randi Marie Selmer, Jannicke Igland, Inger Ariansen, Haakon Eduard Meyer

**Affiliations:** 1 Research Department, Stavanger University Hospital, Helse Stavanger HF, Stavanger, Norway; 2 Department of Chronic Diseases, Norwegian Institute of Public Health, Oslo, Norway; 3 Department of Global Public Health and Primary Care, University of Bergen, Bergen, Vestland, Norway; 4 Department of Health and Caring Sciences, Western Norway University of Applied Sciences, Bergen, Vestland, Norway; 5 Department of Physical Health and Ageing, Norwegian Institute of Public Health, Oslo, Norway; 6 Department of Community Medicine and Global Health, University of Oslo, Oslo, Norway

**Keywords:** Myocardial Infarction, Coronary Artery Disease, Epidemiology, Stroke

## Abstract

**Aims:**

We aimed to study time trends of acute myocardial infarction (AMI) and stroke incidence rates among immigrant groups living in Norway, with a special focus on immigrants from South Asia and former Yugoslavia.

**Methods:**

All incident AMI and stroke events were identified in Norwegian residents aged 35–79 years during 1996–2019 using hospital and cause of death registry data. A 3-year wash-out period was used to identify incident events. Thus, cases were counted from 1999 onwards. We calculated annual age-standardised incidence rates using direct standardisation. Poisson regression was used to calculate the average annual change in incidence rates of AMI and stroke and to study differences between immigrant groups and the Norwegian-born population.

**Results:**

Age-standardised incidence rates of AMI were higher in immigrants from South Asia and former Yugoslavia than in the Norwegian-born population. For Norwegian-born men and women, and former Yugoslavian women, the annual age-standardised AMI incidence rates declined over the study period by 2.4%, 2.0% and 2.3%, respectively. South Asian men and women and former Yugoslavian men did not experience such a decline, although there was an apparent decline in the last 3 years of the period for South Asian men. For former Yugoslavian men, this resulted in increasing differences compared with Norwegian-born men. For stroke, all these groups had declining trends in incidence rates, and former Yugoslavian women had the strongest decline of 4.3% annually.

**Conclusion:**

During 1999–2019, immigrants from South Asia and former Yugoslavia did not experience the same beneficial decline in AMI incidence as the Norwegian-born population. However, both immigrant groups experienced similar or larger declines in the incidence of stroke as Norwegian-born men and women.

**WHAT IS ALREADY KNOWN ON THIS TOPIC**

- Immigrants from South Asia and former Yugoslavia have a high risk of cardiovascular disease (CVD) compared with Norwegian-born individuals.
- The Norwegian majority population has experienced significant declines in the incidence and mortality of CVD, but the trends have never been studied in the immigrant population in Norway.

**WHAT THIS STUDY ADDS**

- Immigrants from former Yugoslavia and South Asia had higher incidence rates of acute myocardial infarction (AMI) than the Norwegian-born population, which persisted throughout the study period.
- South Asian and former Yugoslavian immigrants experienced similarly large declines in stroke rates as that of the Norwegian-born majority population.

**HOW THIS STUDY MIGHT AFFECT RESEARCH, PRACTICE OR POLICY**

- Our study urgently calls for targeted cardiovascular prevention efforts aimed at the immigrant population to reduce the risk of AMI.

## Introduction

 Time trends in cardiovascular disease (CVD) provide information about how environmental and societal factors affect the susceptibility of diseases over time, and how well the diseases are managed and prevented. In Norway and other developed countries, cardiovascular mortality has decreased over the last decades due to improvements in prevention and treatment.^1^ The incidence rates of acute myocardial infarction (AMI) and stroke have also declined in the Norwegian population since 2001 and are still declining.^2^,^5^ We previously found that immigrants from South Asia and the former Yugoslavia had a higher cardiovascular risk than Norwegian-born individuals and that the risk of CVD varied substantially between immigrant groups in Norway.^6^ Yet, the trends in AMI and stroke among immigrants in Norway have never been studied. It is therefore unknown whether immigrant groups have experienced similar favourable trends in the incidence rate and mortality of CVD as the Norwegian majority population, and if the previously observed differences between immigrant groups and the Norwegian-born population in Norway have remained, decreased or increased since this was last studied.

Immigrants and individuals born in Norway to immigrant parents constitute a substantial share of the Norwegian population (20%).^7^ Immigrants in Norway are young, but this is expected to change with a growing number of older immigrants in Norway in the future.^8^ The risk of CVD and other non-communicable diseases increases with age. It is therefore progressively important to understand the epidemiology of CVD in the immigrant population and to determine whether observed cardiovascular trends apply to all groups in the Norwegian population.

The aims of this study are twofold: (1) to report national time trends of AMI and stroke incidence rates for immigrant groups in Norway and (2) to provide estimates of the differences between immigrant groups and the Norwegian-born population.

## Methods

### Data sources

This paper is a substudy in the project *A life-course approach to prevent noncommunicable diseases in an aging population - NCDNOR*. Noncommunicable diseases in Norway (NCDNOR) is a research project aiming at establishing new knowledge in the prevention of noncommunicable diseases by combining noncommunicable disease endpoints across somatic disciplines, examining effects of socioeconomic circumstances, health behaviours, biological markers and mental health throughout the life-course.^9^ We included hospital data from two sources in the NCDNOR project: (1) historical hospital data reused from a previous research project using data from the Patient Administrative System from all Norwegian hospitals covering the years 1996–2008^10^ and (2) the Norwegian Patient Registry providing hospital registry data for the period 2008–2019. Combined, these two sources gave complete hospital data for the years 1996–2019. The hospital data were linked to the Norwegian Cause of Death Registry providing information about fatal cardiovascular events occurring outside of the hospital, and to data from Statistics Norway. Statistics Norway provided demographic information about region of birth, immigrant category, sex, annual population register status and year of birth.

### Study population

We included registered residents in Norway aged 35–79 years and restricted to a minimum of 3 years of residency. This included individuals born in Norway as well as immigrants born in 15 different regions of the world (see online supplemental table 1 for grouping of countries into regions). Immigrants were defined as individuals born outside of Norway with two foreign-born parents and four foreign-born grandparents according to the definition of Statistics Norway. Norwegian-born individuals were defined as those born in Norway, including those born to immigrant parents, who constituted only 0.4% of the Norwegian-born group in 2020 among those aged 35–79 years.

### Definition of endpoints

An incident AMI event was defined as either an inpatient hospitalisation with AMI as main or secondary diagnosis (ICD-9: 410 and ICD-10: I21, I22) or death with ischaemic heart disease (IHD) as underlying cause of death (ICD-9: 410–414 and ICD-10: I20–I25) with no previous hospitalisation for AMI in the three preceding years. An incident event of stroke was defined as either an inpatient hospital admission where stroke was the main or secondary diagnosis (ICD9: 430–434, 436 and ICD10: I60–I61, I63–I64 except I63.6) or death with stroke as the underlying cause of death, with no previous hospitalisation for stroke in the three preceding years. However, stroke as a secondary diagnosis was only included when the primary diagnosis was not stroke sequelae (ICD9: 438 and ICD10: I69) or rehabilitation (ICD9: V57 and ICD10: Z50.80, Z50.89) to avoid counting false positive cases of stroke.^11^ Both for AMI and stroke, the choice of a fixed 3-year look-back period was done to balance the possibility of identifying incident events, available period for analysis and statistical power. A short look-back period increases the probability of including recurrent events, while a longer period would require exclusion of individuals with shorter residency and leave a shorter time period for analyses, which would give reduced statistical power.

### Statistical analyses

We calculated age-standardised incidence rates by the direct standardisation method, with 5-year age strata using the Norwegian population of 2001 as standard population. The denominator for the rates included all registered residents in Norway on 1 January of the actual year, who were also registered residents for the three preceding years and did not experience an event (AMI or stroke) during the three preceding years. Age-standardised incidence rates were calculated for three periods within the study period (1999–2005, 2006–2012 and 2013–2019) for all immigrant groups. We divided into three periods to provide an overview of the trends for all immigrant groups in Norway, while also preserving enough power for it to be meaningful. We also calculated annual incidence rates for Norwegian-born individuals, and South Asian and former Yugoslavian immigrants stratified by sex. These groups were chosen due to their higher risk of CVD compared with Norwegian-born individuals.^6^

We estimated incidence rate ratios (IRRs) for incident AMI and stroke events in the immigrant groups with Norwegian-born individuals as reference using Poisson regression. Groups with IRRs statistically significantly higher than one were considered to have an excess risk compared with Norwegian-born individuals. We adjusted for age in 5-year age groups and for calendar year as a continuous variable. When the goodness-of-fit test was significant for the Poisson regression, negative binomial regression was used instead. IRRs for relative difference between the immigrant groups and the Norwegian-born reference population were calculated for two periods within the study period (before and after 2010) to re-examine relative differences in incidence rates of AMI and stroke that were previously published using data from 1994 to 2009.^6^ We also examined interactions between calendar year and ethnic groups using Poisson regression to test for differences in trend.

Average annual change in incidence rate (IRR-1) with 95% CIs was estimated within each of the selected groups using Poisson regression adjusted for age in 5-year age groups with calendar year as a continuous variable.

Stata V.16 was used for all these analyses.

### Sensitivity analyses

We examined non-linear trends in age-standardised AMI and stroke rates using Joinpoint Regression Analyses^12^ using the Joinpoint software (Joinpoint Regression Program, V.5.0.2, May 2023; Statistical Research and Applications Branch, National Cancer Institute in the USA). Based on permutation testing, the joinpoint regression identifies points (‘joinpoints’) where the trends change, and then fits the simplest joinpoint model that the data allow.^12^

## Results

During 1999–2019, over 3.5 million individuals aged 35–79 years in Norway were at risk of having an incident AMI (n=3 636 847, 14% immigrants) or stroke (n=3 618 345, 13% immigrants). A total of 209 961 incident AMIs and 149 750 incident stroke events were observed during the 21-year-long study period. Individuals could experience more than one incident event if more than 3 years passed between events, but most events were first incident events (93% for AMI and 94% for stroke). Age-standardised incidence rates of AMI and stroke for three periods are shown for selected immigrant groups in Norway in tables1  2, while complete tables including all immigrant groups can be found in online supplemental tables 2 and 3. In 2013–2019, the age-standardised incidence rates of AMI were highest in immigrants from South Asia (949 per 100 000 person-years among men and 377 per 100 000 person-years among women), followed by immigrants from former Yugoslavia (686 among men and 242 among women) and Central Asia (628 among men and 220 among women) (table 1 and online supplemental table 2). For stroke, there were less marked differences in age-standardised incidence rates than for AMI, and more uncertainty in the rates due to fewer endpoints (table 2 and online supplemental table 3).

**Table 1:** Age-standardised incidence rates of acute myocardial infarction, for men and women from different birth regions for three periods within the study period

Birth region	1999–2005		2006–2012		2013–2019	
	Cases (n)	Age-standardised incidence rates (95% CI) per 100 000 PY	Cases (n)	Age-standardised incidence rates (95% CI) per 100 000 PY	Cases (n)	Age-standardised incidence rates (95% CI) per 100 000 PY
Men, n*=1 742 055						
Norwegian, n=1 561 709	49 416	740 (734 to 746)	47 338	651 (645 to 657)	41 865	498 (494 to 503)
Western European, n=69 846	904	713 (663 to 762)	1 026	595 (556 to 635)	1 144	450 (422 to 477)
Eastern European, n=77 170	162	865 (733 to 998)	195	623 (517 to 730)	588	435 (372 to 499)
Former Yugoslavian, n=14 355	188	758 (627 to 890)	314	744 (649 to 838)	386	686 (607 to 767)
South Asian, n=18 975	341	907 (767 to 1045)	540	1062 (934 to 1190)	737	949 (871 to 1028)
Women, n=1 703 168						
Norwegian, n=1 570 396	22 538	290 (287 to 294)	19 603	252 (249 to 256)	17 346	197 (194 to 200)
Western European, n=56 534	414	238 (213 to 259)	422	213 (193 to 234)	357	145 (130 to 160)
Eastern European, n=47 318	39	242 (165 to 320)	58	175 (121 to 229)	134	173 (135 to 211)
Former Yugoslavian, n=13 118	75	359 (271 to 446)	127	380 (310 to 449)	108	242 (192 to 293)
South Asia, n=15 802	58	359 (239 to 479)	145	500 (398 to 603)	195	377 (313 to 440)

* n=number of individuals in the age range 35–79 years who were included in the population at risk at least 1 year during the study period.

PYperson-years

**Table 2:** Age-standardised incidence rates of stroke, for men and women from different birth regions for three periods within the study period

Birth region	1999–2005		2006–2012		2013–2019	
	Cases (n)	Age-standardised incidence rates (95% CI) per 100 000 PY	Cases (n)	Age-standardised incidence rates (95% CI) per 100 000 PY	Cases (n)	Age-standardised incidence rates (95% CI) per 100 000 PY
Men, n*=1 742 192						
Norwegian, n=1 561 811	28 617	433 (428 to 439)	27 987	391 (387 to 396)	26 889	322 (318 to 326)
Western European, n=69 852	492	420 (381 to 460)	595	366 (334 to 399)	785	331 (306 to 356)
Eastern European, n=77 186	87	475 (375 to 575)	90	322 (241 to 403)	287	267 (212 to 322)
Former Yugoslavian, n=14 361	80	374 (280 to 468)	126	370 (298 to 443)	140	285 (230 to 341)
South Asian, n=18 982	112	444 (315 to 572)	148	410 (313 to 507)	237	375 (319 to 432)
Women, n=1 702 546						
Norwegian, n=1 569 797	21 854	283 (279 to 286)	19 182	248 (245 to 252)	17 699	203 (200 to 206)
Western European, n=56 515	439	254 (231 to 278)	430	222 (200 to 243)	415	170 (154 to 187)
Eastern European, n=47 313	42	221 (151 to 292)	74	202 (146 to 257)	165	195 (156 to 235)
Former Yugoslavian, n=13 119	75	385 (292 to 477)	94	291 (229 to 353)	89	206 (159 to 253)
South Asian, n=15 802	53	288 (182 to 395)	88	305 (225 to 386)	125	234 (184 to 284)

* n=number of individuals in the age range 35–79 years who were included in the population at risk at least 1 year during the study period.

PYperson-years

### Relative differences between groups before and after 2010

**Figure 1:**
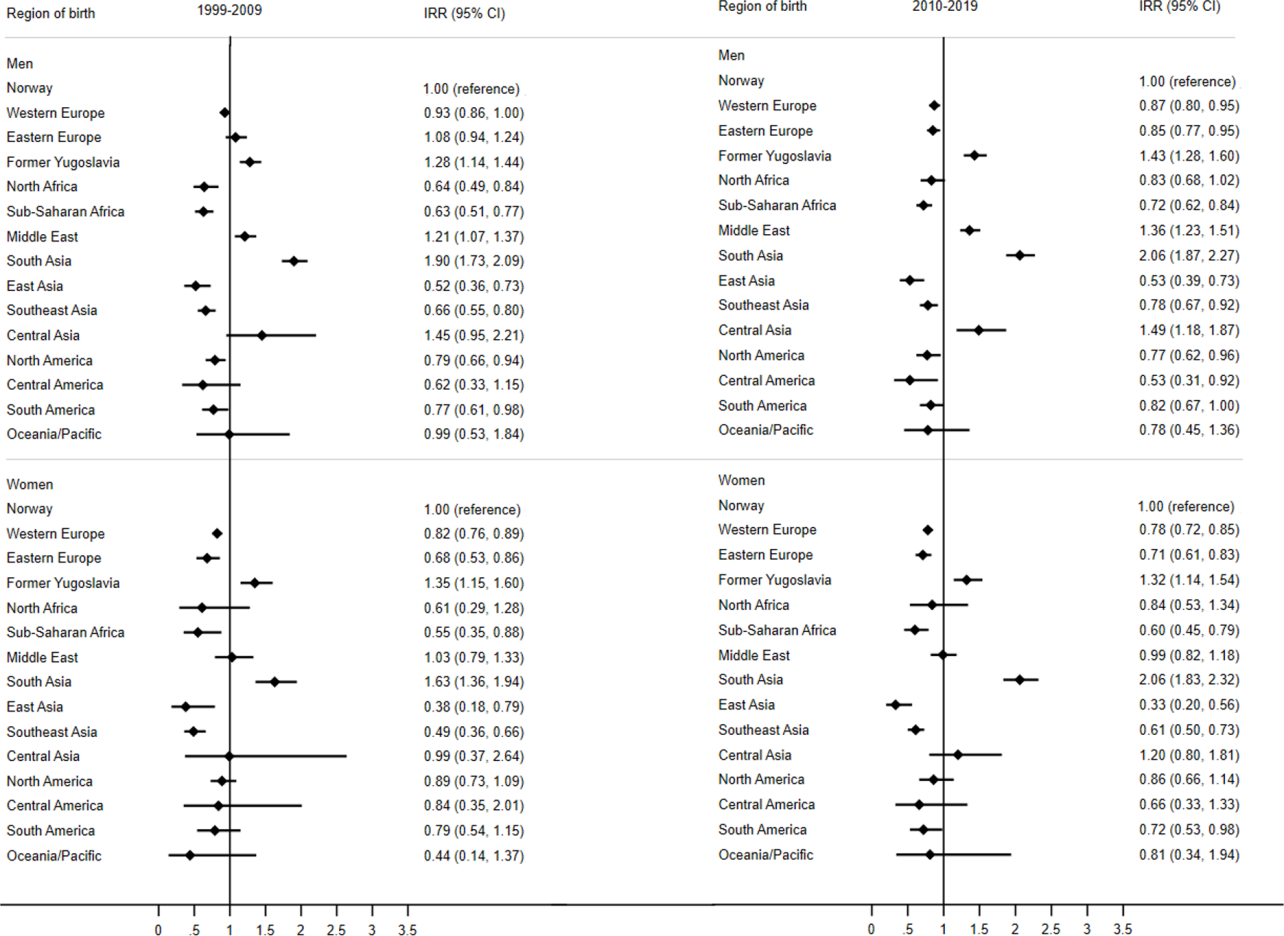
Forest plots of incidence rate ratios (IRRs) for acute myocardial infarction in immigrant men and women aged 35–79 years relative to Norwegian-born individuals, before and after 2010.

IRRs for the differences in incidence rates between immigrant groups and the Norwegian-born reference group before and after 2010 are shown in figures1  2 for AMI and stroke, respectively. South Asian men and women had the highest excess risk of AMI relative to Norwegian-born individuals, with 90% excess risk in men and 63% excess risk in women for the years 1999–2009 (figure 1). For 2010–2019, the estimates were slightly higher, especially for women, with 106% excess risk in both men and women. The IRRs for former Yugoslavian men and women showed that this group also had an excess risk of AMI compared with Norwegian-born individuals both before and after 2010 (figure 1). For men from former Yugoslavia, the estimates increased from an excess risk of 28% in the first period to an excess of 43% in the last period. The estimates remained constant over the two periods for former Yugoslavian women (35% and 32% excess risk). Middle Eastern men also had higher incidence rates of AMI compared with the Norwegian-born men over both periods. Immigrants from East Asia had low risk of AMI in both periods (figure 1). Immigrants from Sub-Saharan Africa also had lower incidence of AMI compared with Norwegian-born individuals in both sexes and both periods. Low risk was also found in immigrant men from North Africa for 1999–2009 (figure 1).

**Figure 2:**
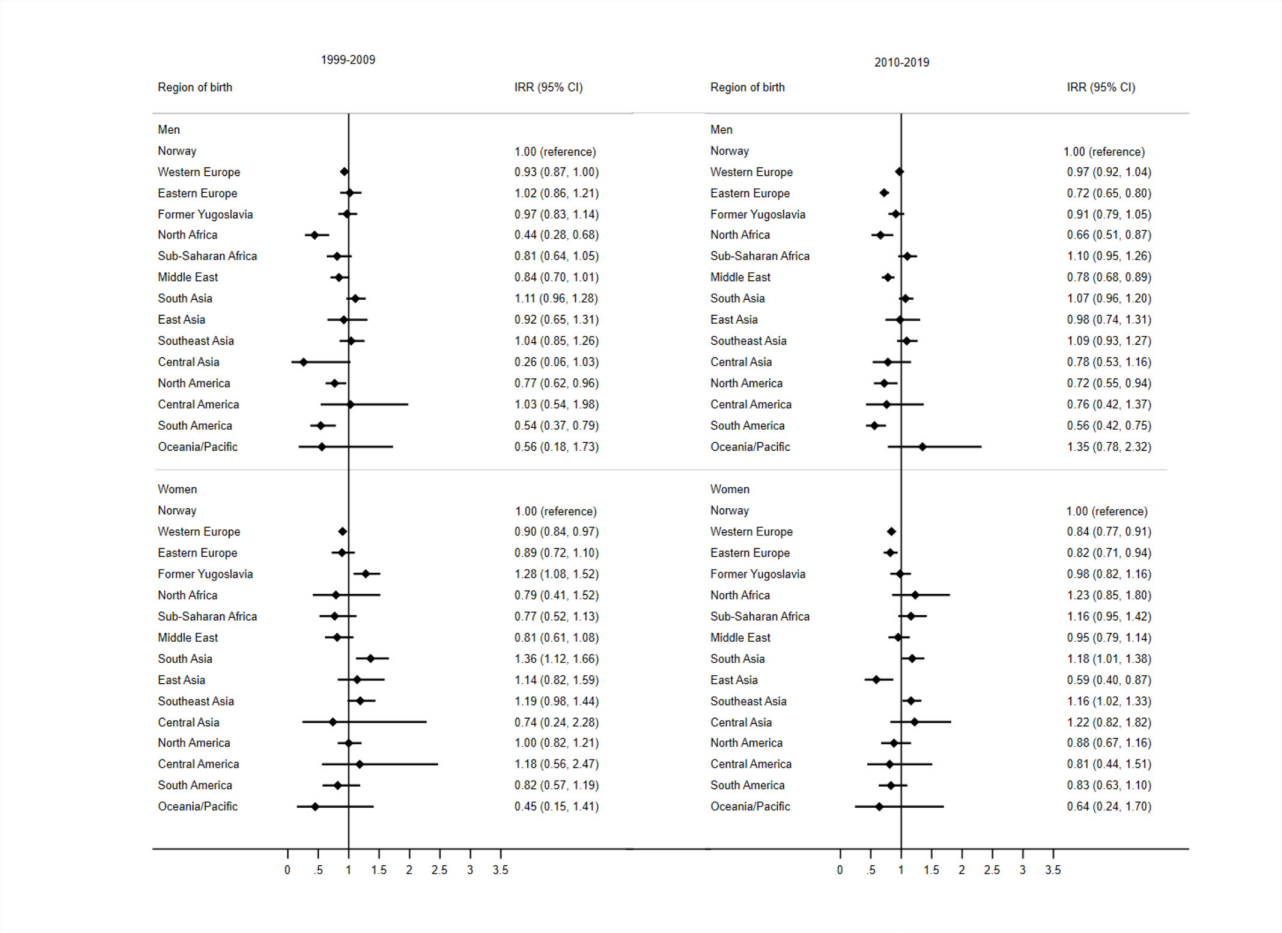
Forest plots of incidence rate ratios (IRRs) for stroke in immigrant men and women aged 35–79 years relative to Norwegian-born individuals, before and after 2010.

Differences in risk of stroke for immigrant groups relative to the Norwegian-born individuals were less pronounced than for AMI (figure 2). Among men, no immigrant group had an excess risk of stroke compared with the Norwegian-born men. Immigrants from North Africa, North America and South America, on the other hand, had lower risk of stroke than Norwegian-born men, and this remained in both periods (figure 2). Immigrant men from Eastern Europe and men from the Middle East had reduced risk in the last period (figure 2).

Among women, a few immigrant groups had an excess risk of stroke compared with Norwegian-born women. Women from former Yugoslavia had 28% excess risk of stroke and women from South Asia had 36% excess risk of stroke compared with Norwegian-born women for the years 1999–2009 (figure 2). For the last period 2010–2019, there was no longer an excess risk in former Yugoslavian women, but a borderline excess risk of 18% remained in South Asian women. We also found a borderline excess risk of 16% in Southeast Asian women in 2010–2019. East Asian immigrant women, however, had 41% lower risk of stroke in the last period compared with Norwegian women. Immigrant women from Western and Eastern Europe also had 16% and 18% lower risk for the last part of the study period, respectively (figure 2).

### Trends in AMI

**Figure 3:**
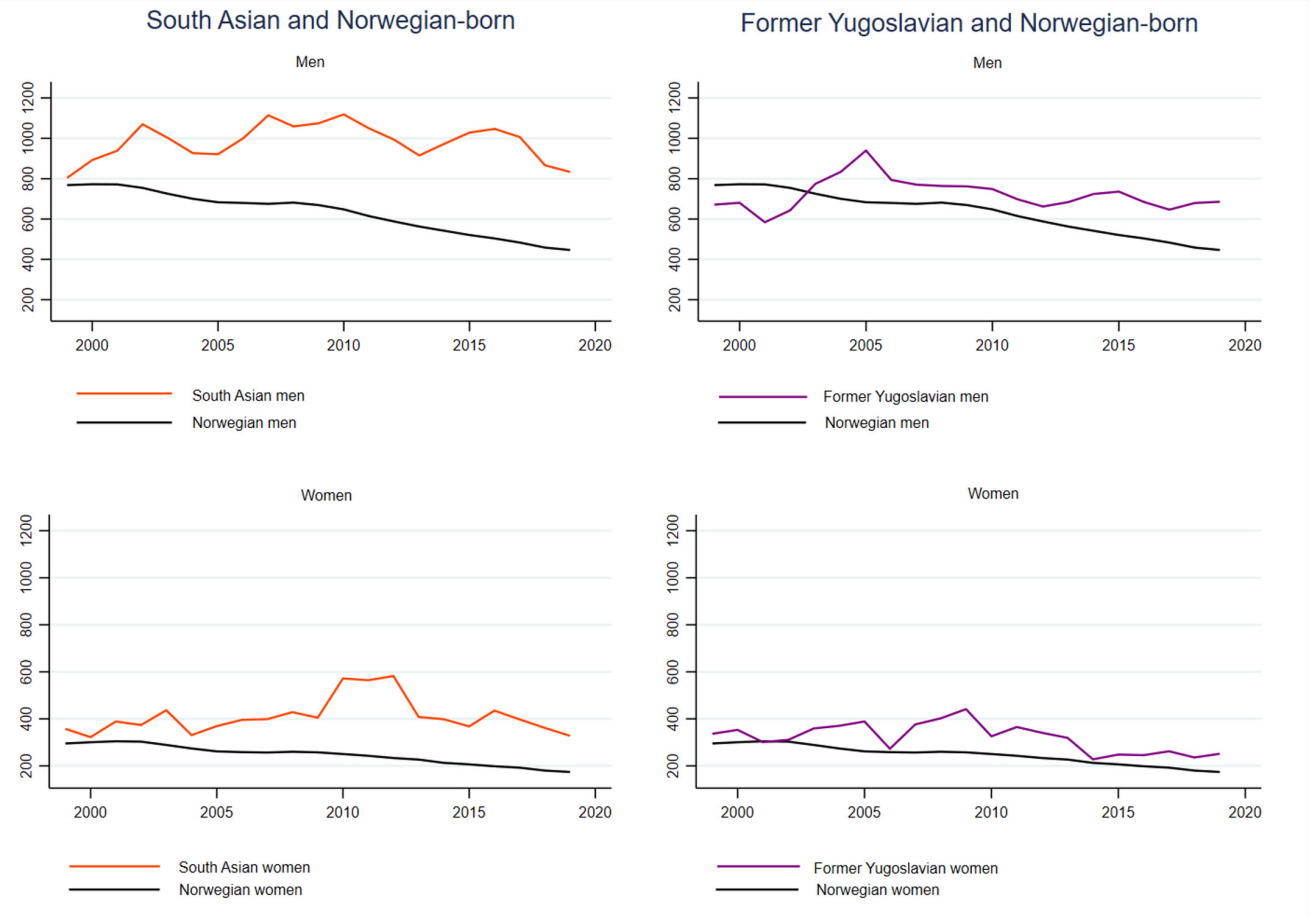
3-year moving averages of age-standardised acute myocardial infarction incidence rates, men and women aged 35–79 years. Norwegian-born individuals and immigrants from South Asia and former Yugoslavia.

For men and women born in Norway, South Asia and former Yugoslavia, the 3-year moving averages of the annual age-standardised incidence rates for AMI and stroke are depicted in figures3  4, respectively. Annual age-standardised incidence rates with CIs included are shown in online supplemental figures 1 and 2.

**Figure 4:**
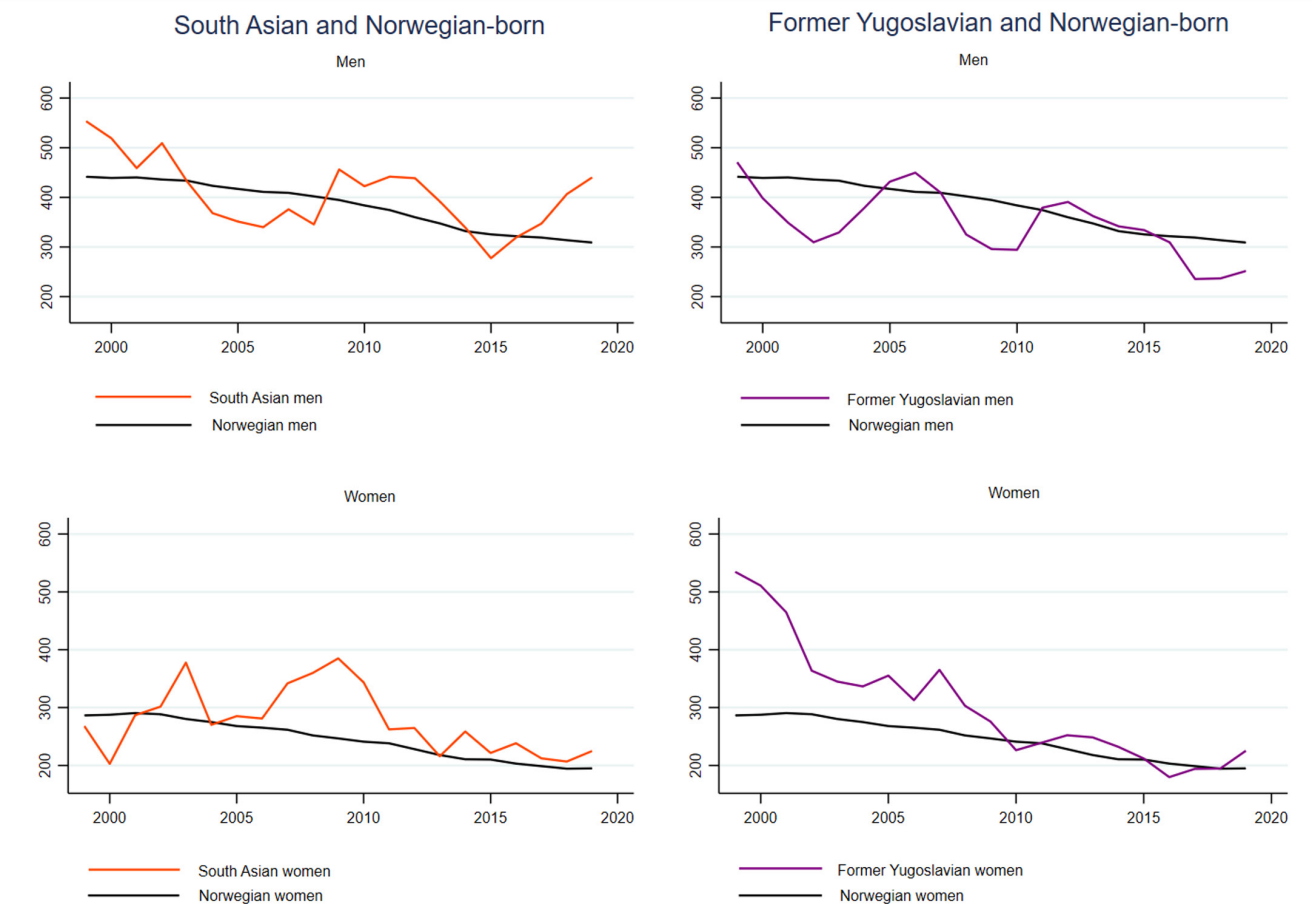
3-year moving averages of age-standardised stroke incidence rates, men and women aged 35–79 years. Norwegian-born individuals and immigrants from South Asia and former Yugoslavia.

As expected, we found a marked and steady decline in incidence rates of AMI among Norwegian-born men and women (figure 3) with an annual average risk reduction of 2.4% in men and 2.0% in women (online supplemental table 4). The age-standardised incidence rates of AMI for South Asians and former Yugoslavians generally showed a flatter trend over the study period than among Norwegian-born individuals (figure 3 and online supplemental figure 1). An exception was former Yugoslavian women who had a significant annual decline of 2.3% (online supplemental table 4).

Interaction terms between calendar year and birth region revealed significantly weaker declines in AMI rates in men from former Yugoslavia (p interaction=0.043) than in their Norwegian-born counterparts (online supplemental table 4). In other words, there were widening differences in AMI incidence between men from former Yugoslavia and Norwegian-born men. Men from Eastern Europe were found to have stronger declines (p interaction=0.004) in AMI incidence rates than Norwegian-born men over the study period (online supplemental table 4).

### Trends in stroke

As for AMI, we found a steady decline in annual age-standardised stroke rates among Norwegian-born men and women with average annual risk reductions of 1.6% in men and 1.9% in women (figure 4 and online supplemental table 5). Contrary to what we found for AMI, South Asian and former Yugoslavian men and women also had declining age-standardised stroke rates (figure 4 and online supplemental table 5). Former Yugoslavian women had the largest decline of all these groups including Norwegian-born individuals, with an average annual change of −4.3% (online supplemental table 5). Furthermore, the interaction test showed that this decline was larger than the decline in Norwegian-born women (p interaction=0.020). Thus, the differences in age-standardised stroke rates between former Yugoslavian and Norwegian-born women diminished over the study period, resulting in similar rates for the two groups in the last part of the study period (figures2  4 and table 2). Men from Eastern Europe had stronger declines in stroke incidence rates than Norwegian-born men (p interaction=0.001) (online supplemental table 5).

### Sensitivity analyses

The joinpoint regression analyses did not identify any changes in trends of AMI incidence rates for South Asian women, nor former Yugoslavian men or women. Similarly for stroke, no changes in trends for the age-standardised stroke rates for South Asian and former Yugoslavian immigrant men and women were identified. It was therefore acceptable to assume linearity in these trends, and the average annual change estimates from Poisson regression were considered valid (online supplemental tables 4 and 5).

For South Asian men, however, the joinpoint regression identified a significant change in trend of AMI incidence rates in 2016. Although the Poisson regression found a significant annual decline of 1.4% for South Asian men over the whole period (online supplemental table 4), the results from the joinpoint regression suggested that this mostly applied to the last 3 years of the study period. A further test of trend using Poisson regression supported this. No significant effect of calendar year was found for the years before 2016, but from 2016 onwards an annual decline of 12% (IRR, 0.88, 95% CI 0.81 to 0.96, p=0.004) was identified.

## Discussion

The previously described excess risk of AMI in immigrants from South Asia and former Yugoslavia compared with Norwegian-born individuals remained until the end of the study period in 2019.^6^ This is the first study to examine time trends in the incidence of AMI and stroke among immigrant groups living in Norway. Our study revealed that some immigrant groups (South Asian women and former Yugoslavian men) did not experience the same beneficial decline in the incidence of AMI rates as has previously been reported for the Norwegian majority population.^2 3^ An uplifting result, however, was the finding that both South Asian and former Yugoslavian immigrants experienced declining trends in stroke rates over the study period, similar to (and for former Yugoslavian women stronger than) the trends in the Norwegian-born population.

The steady decrease in AMI incidence rates among Norwegian-born individuals combined with the lack of decrease in former Yugoslavian men resulted in widening differences between these two groups. This was evident both from the annual age-standardised incidence rates and from the interaction that was found between calendar year and ethnic group showing a statistically larger decrease in the Norwegian-born group. It was also reflected by higher relative risk estimates for the latest part of the study period versus before 2010 for former Yugoslavian men relative to Norwegian-born men. The causes behind these widening differences in AMI are not clear. However, it does reveal a persistent need to improve the preventive efforts aimed at the immigrant population in Norway, preferably with a special focus on immigrants from South Asia and former Yugoslavia. On the positive side, the apparent decline in age-standardised AMI rates in South Asian men for the last 3 years of the study period could be an indication that the trend is starting to change for the better for South Asian immigrants. Still, this 3-year decline might be temporary and should be interpreted with caution. It will be of particular interest to examine this potential decline in the future, to see if it continues.

A few studies have examined trends in cardiovascular outcomes in different ethnic groups living in high-income countries. The results vary for different immigrant groups and the different host countries. While some studies find declining rates for all included groups,^13^,^15^ other studies find that some immigrant groups have stable or increasing trends.^16^,^18^ In California, declining trends of AMI were found in all groups (Hispanic and non-Hispanic groups: Asian or Pacific Islander, black and white) for the years 2000–2014.^13^ In England and Wales, however, not all immigrant groups had the same beneficial decline as the majority population in IHD and stroke mortality during 1979–2003.^16^ For immigrants from Pakistan, Bangladesh and Poland, the disparities in coronary mortality increased over the period.^16^ In New Zealand, all studied groups (European, Mäori, Pacific, Indian and other Asian) had declining trends in IHD during 2006–2015.^15^ Similarly, a Dutch study found that most major migrant groups in the Netherlands had declining trends in incidence of AMI like the majority population.^18^ It is challenging to compare results between studies that examine trends in CVD for immigrant groups in different host countries, since the results may vary and contradict due to different compositions of immigrant groups and to differing risks in the reference populations.

Many factors including socioeconomic conditions, individual and lifestyle factors, experiences before, during and after migration, characteristics of the country of origin and the country of destination all influence the differences in health between host populations and immigrants. For the South Asian group, our finding of a high risk of AMI confirms and adds to the international literature on a high risk of IHD in the South Asian diaspora.^19^ Numerous studies have been conducted aiming to disentangle the reasons for their increased risk of CVD compared with populations of European descent, without yielding one clear answer.^19 20^ There is little doubt, however, that the high prevalence of type 2 diabetes in this group and the cardiovascular risk it poses through glycosylation and dyslipidaemia plays an important role.^19 21^ It is also important to remember that known cardiovascular risk factors apply to South Asians as to other ethnic groups, and that the focus on traditional risk factors is key for prevention in all populations.^19^ Access to healthcare and health-seeking behaviour are also factors that can affect the development of cardiovascular risk over time in the different groups. One Norwegian study looking at the management of type 2 diabetes among immigrants in Norway found indications of equal access to treatment, but the achievement of treatment goals was not equally met among non-Western minority groups compared with the Western reference group.^22^ The reasons for this are not clear.

Interestingly, we found a lower risk of AMI in immigrants from East and Southeast Asia as well as Sub-Saharan Africa and North Africa (non-significant among North African women) compared with the Norwegian-born group. These results confirmed our previous findings.^6^ Yet, the reasons for lower risk among these groups are not well understood. To some degree, the variation in risk of AMI versus stroke among the Asian subgroups somewhat reflects patterns in the epidemiology of CVD mortality in the Asian region.^23^ In Asia, IHD has been found to be the most dominant cause of CVD deaths in Central, Western and Southern parts of the region, while stroke is the most dominant cause in Eastern and Southeastern Asia.^23^ This corresponds with the lower risk of AMI in East and Southeast Asia versus South and Central Asia in our study, yet generally not a lower risk of stroke in these groups.

A review on the risk of IHD and stroke among immigrant populations living in high-income countries found that longer duration of residence in the host country was associated with increased relative risks of IHD and stroke in most of the studies.^24^ This corresponds with our results for AMI in South Asian women and former Yugoslavian men. We did not control for duration of residence in our analyses, but duration of residence increased for these groups in our data over the study period. Several studies have found that the health in immigrant populations often converges towards the health in the host population with increased length of stay.^25^,^27^ Convergence in health has typically been described when there is an initial health advantage in the immigrant population, in accordance with the ‘healthy immigrant effect’.^28^ The initial health advantage in the immigrant population often dissolves after time of residence in the host country, sometimes termed ‘negative acculturation’, or ‘negative/unhealthy assimilation’.^25 27^ Although the higher risk of AMI in some of the immigrant groups does not support a healthy immigrant effect, the prevailing differences in South Asian and former Yugoslavian immigrants are in line with a negative acculturation effect. A healthy immigrant effect in all-cause mortality was previously found in newly arrived immigrants to Norway, but the mortality increased with length of stay—also supporting a worsening in health over time.^25^ Immigrants who migrated due to work or education had a stronger survival advantage than refugees.^25^ The healthy immigrant effect is often explained by immigrants being selectively young and healthy (selective in-migration).^29^ Another aspect is the ‘salmon bias’ hypothesis, where less healthy people return to their home country before they die (selective out-migration).^29^ A similar effect could apply to CVD endpoints if unhealthy immigrants return to their home country and experience CVD events there. This hypothesis has generally gained little scientific support but was recently found to be partly responsible for lower mortality rates among immigrants in Italy.^30^

Men from Eastern Europe (the largest immigrant group in Norway) had stronger declines in both AMI and stroke incidence rates than Norwegian-born men, contradicting a convergence in health for this group. The ‘healthy immigrant effect’ could possibly be stronger for this group since immigrants from Eastern Europe are mostly young immigrants who have come to work in Norway, especially after the expansion of the European Union in 2004.^31^ Although a substantial share of Polish working migrants have settled in Norway,^31^ many migrants from Eastern Europe emigrated during 2017–2019.^32^ Some can also have emigrated without it being registered. Although we censored individuals in our analyses when people were no longer registered Norwegian residents, the mobility of immigrants to and from Eastern Europe, where the geographical distance is short, could possibly have contributed to maintaining a selectively healthy group of immigrants or caused cardiovascular events to go unnoticed in Norwegian registry data. In 2016, Polish immigrants reported a relatively high smoking prevalence and reported two times as often about anxiety/depression than the Norwegian majority population.^33^ Thus, this group is perhaps not healthier than other groups, and selection mechanisms could partly explain the mostly lower incidence rates of AMI and stroke found in this immigrant group, and the strong decline in both endpoints among Eastern European men.

While men from former Yugoslavia did not experience declining trends in AMI incidence, the former Yugoslavian women experienced a similar decline in AMI rates as Norwegian-born women. The excess risk compared with Norwegian-born women therefore remained constant for the whole study period in former Yugoslavian women (35% before 2010 and 32% after). Immigrants from former Yugoslavia in Norway are largely individuals who fled the Balkan wars in the beginning of the 1990s, with refugees from Bosnia and Hercegovina as one large subgroup within this group.^34^ Immigrants from Bosnia and Herzegovina are highly integrated in the Norwegian society in terms of participating in work and education.^34^ On average, this group is highly educated and employed to about the same degree as the majority population in Norway.^34^ We do not fully understand the reasons for an excess risk of AMI in former Yugoslavians, but war traumas before migration and post-traumatic stress disorder (PTSD) could possibly be relevant. A Swedish study found increased risk of CVD and PTSD among refugees from Balkan wars living in Sweden.^35^ PTSD is linked to cardiovascular health through different mechanisms only partly understood and includes indirect links through unhealthy behaviours.^36^ Based on data from Norwegian health surveys conducted 1994–2003, we previously found that immigrants from former Yugoslavia had high levels of cardiovascular risk factors and the highest Framingham risk score among 10 other immigrant groups and Norwegian-born individuals.^37^ The levels of risk factors may have changed for this group since then, supported by decreasing numbers of self-reported daily smokers among immigrants from Bosnia and Hercegovina between 2005/2006 and 2016, from 36% to 23% of daily smokers for men and from 31% to 19% for women.^38 39^ The reduction in smoking habits is positive in itself, but could also reflect general improvements in cardiovascular risk factors which may have contributed to the declining trends in age-standardised rates of stroke in former Yugoslavian immigrants, and declining AMI rates for women in this group. We previously found a 28% increased risk of stroke in former Yugoslavian immigrant men,^6^ but this was not found in the present study. It could be that former Yugoslavian men have had an excess risk limited to the first years of the previous study (1994–1999) as these years were not included in the present study.

The declining rates of stroke in immigrants from South Asia and former Yugoslavia were encouraging. The reason for decreasing trends in rates of stroke but not AMI in these immigrant groups is unclear. Although AMI and stroke largely share the same risk factors, it is possible that risk factors more strongly associated with stroke than AMI have had a different development in these immigrant groups. Cholesterol has, for example, been found to be more strongly associated with the risk of IHD, while blood pressure is more strongly linked to stroke.^40^ We previously found that immigrants from South Asia had an unhealthy lipid profile and higher prevalence of diabetes, but not higher blood pressure values than Norwegian-born individuals, which could contribute to explain why the relative risk of AMI for South Asians is markedly higher than the relative risk of stroke in the present study.^21^ Based on the large differences in especially AMI, health authorities, local government and so forth should improve and target health promotion programmes in order to reach the groups with the highest incidence.

### Strengths and limitations

An important strength is the nationwide register-based data that enabled us to study the whole population in Norway over a 21-year-long study period. This strengthens the external validity of the results. This study provides novel information about the development of acute cardiovascular endpoints in a growing and ageing immigrant population in Norway.

Despite the large and comprehensive dataset, there were few yearly cases of AMI and stroke in South Asian and former Yugoslavian men and women, especially in the first years, which gave fluctuating annual rates and wide 95% CIs.

The validation of AMI diagnoses in the Norwegian Patient Registry was examined in 2020 and deemed adequately complete and correct for research use.^41^ The validity of the stroke diagnosis in the Norwegian Patient Registry has also been deemed valid for epidemiological purposes.^11^ However, when including stroke as a secondary diagnosis in the Norwegian Patient Registry, the validation study found false positive cases that should have been coded as sequelae or rehabilitation after stroke.^11^ We therefore only included stroke as a secondary diagnosis when the primary diagnosis was not stroke sequelae or rehabilitation. There is a possibility that we might miss some true events, but we expect them to be few and randomly distributed across the ethnic groups.

We used a 3-year look-back period to identify incident events. A longer look-back period would have increased the likelihood of only counting first-ever events,^42^ but it would be at the expense of the length of the available study period and statistical power. The length of the look-back period has been found to affect the number of incident AMIs and AMI trend estimates.^42^ However, misclassification caused by a short look-back period is expected to affect all immigrant groups to the same extent and should not introduce any systematic errors when examining trends and differences between groups.

We have studied two acute cardiovascular endpoints, AMI and stroke. This could be considered a limitation as they do not give the full picture of cardiovascular risk. It was not within our scope to study broader endpoints or markers, but we acknowledge that it could have provided a more complete picture of the cardiovascular risk.

It is a limitation that we did not have the possibility to study the impact of lifestyle, behaviour risk factors, healthcare utilisation or biological factors.

## Conclusion

The risk of AMI is still a great concern for immigrants from South Asia and former Yugoslavia living in Norway. The differences in incidence rates of AMI for these two immigrant groups vs the Norwegian-born population remained at the end of the study period. Moreover, the differences increased for former Yugoslavian men. These are important findings that urgently warrant culturally adapted preventive efforts to turn this negative trend.

## supplementary material

10.1136/openhrt-2024-003114 online supplemental file 1

## Data Availability

No data are available.
