# Supplementary material for: Trends in incidence rates of acute myocardial infarction and stroke among immigrant groups in Norway, 1999–2019: the NCDNOR project
Source: Open Heart. 2025 Apr 4;12(1):e003114. doi: 10.1136/openhrt-2024-003114 (PMC11973780; doi:10.1136/openhrt-2024-003114)
Supplement: online supplemental file 1 [file openhrt-12-1-s001.pdf]

Supplementary material to:

Trends in incidence rates of acute myocardial infarction and stroke among immigrant groups in Norway 1999-2019: the NCDNOR project

Kjersti S Rabanal[1], Randi M Selmer[2], Jannicke Igland[3,4], Inger Ariansen[2], Haakon E Meyer[5, 6]

1) Research Department, Stavanger University Hospital, Stavanger, Norway

2) Department of Chronic Diseases, Norwegian Institute of Public Health, Oslo, Norway

3) Department of Health and Caring Sciences, Western Norway University of Applied Sciences, Bergen, Norway

4) Department of Global Public Health and Primary Care, University of Bergen, Bergen, Norway

5) Department of Physical Health and Ageing, Norwegian Institute of Public Health, Oslo, Norway

6) Department of Community Medicine and Global Health, University of Oslo, Oslo, Norway

Supplemental Figure 1. Annual age standardized acute myocardial infarction incidence rates with 95% confidence interval, men and women 35-79 years.

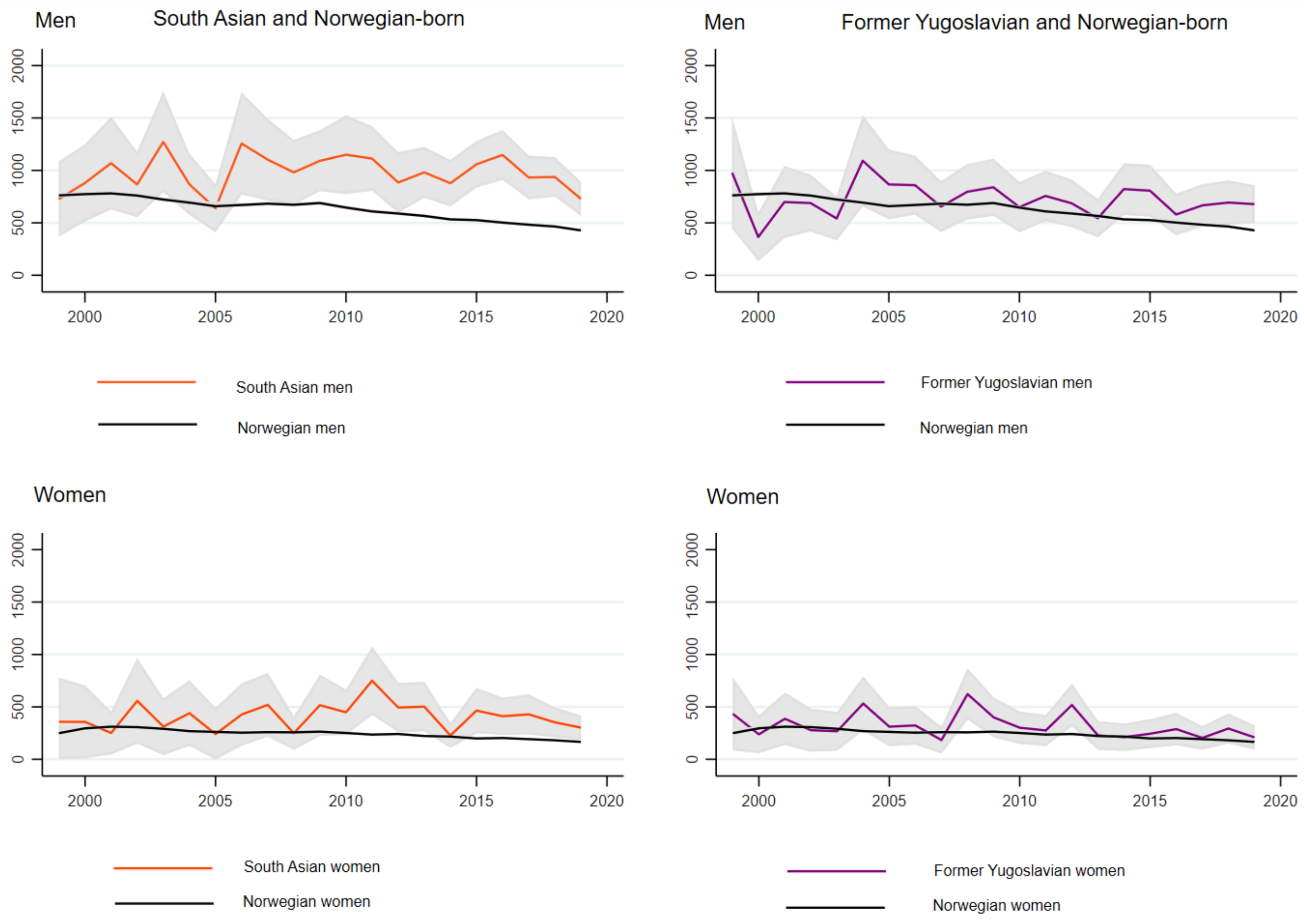

Supplemental Figure 2. Annual age standardized stroke incidence rates with 95% confidence interval, men and women 35-79 years.

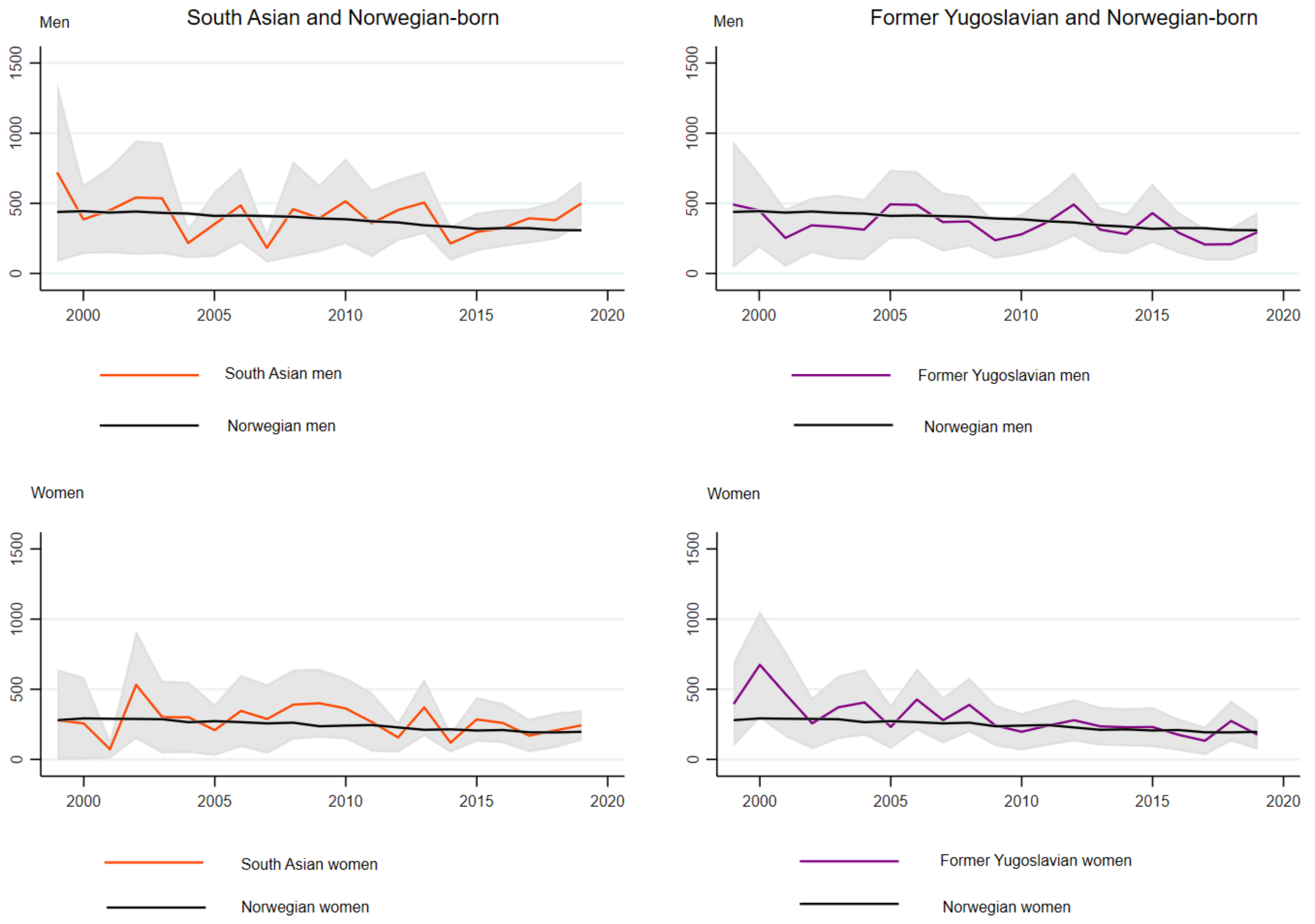

Supplemental Table 1. Birth regions with corresponding countries.

**Regions**	**Countries**
**Norway**	Norway
**Western Europe**	Denmark, Greenland, Finland, Fareo islands, Sweden, Belgium, Andorra, France, Gibraltar, Greece, Ireland, Italy, Malta, Netherlands, Liechtenstein, Luxembourg, Monaco, Portugal, San Marino, Spain, Great Briatin, Switzerland, Germany, Hungary, Austria,<br>Israel, Cyprus, Iceland
**Eastern Europe**	Estonia, Bulgaria, Belarus, Latvia, Poland, Romania, Lithuania, Moldavia, Russia, Ukraine, Hungary, Slovakia, Georgia, Czech Republic
**Former Yugoslavia**	Albania, Croatia, Slovenia, Bosnia-Hercegovina, Macedonia, Serbia, Montenegro, Kosovo
**Middle East**	Turkey, Armenia, Aserbadsjan, Bahrain, The United Arab Emirates, Iraq, Iran, Jordan, Kuwait, Lebanon, Palestine, Qatar, Saudi Arabia, Syria, Yemen, Oman
**North Africa**	Tunisia, Algeria, Egypt, Libya, Morocco, Sudan, Southern Sudan
**Sub-Saharan Africa**	Angola, Botswana, Equatorial Guinea, Ivory Coast, Eritrea, Ethiopia, Djibouti, Gambia, Ghana, Guinea, Guinea-Bissau, Cameroon, Cape Verde, Congo, Liberia, Madagascar, Mauritania, Mauritius, Namibia, Nigeria, Mozambique, Zimbabwe, Rwanda, São Tomé and Príncipe, Senegal, Central African Republic, Sierra Leone, Somalia, South Africa, Burundi, Comoros, Benin, Gabon, Congo-Brazzaville, Kenya, Lesotho, Malawi, Mali, West-Sahara, Niger, Réunion, Seychelles, Swaziland, Chad, Togo, Tanzania, Uganda, Zambia, Burkina Faso
**South Asia**	Bangladesh, Bhutan, Myanmar, Sri Lanka, India, Nepal, Pakistan
**Southeast Asia**	Brunei, Phillipines, Indonesia, Cambodia, Laos, Malaysia, East-Timor, Singapore, Thailand, Vietnam
**East Asia**	Taiwan, Hongkong, Japan, China, North-Korea, South-Korea, Mongolia, Macao
**Central Asia**	Afghanistan, Kasakhstan, Tadsjikistan, Turkmenistan, Kirgisistan, Uzbekistan
**North America**	Canada, USA
**Central America**	Cayman Islands, Costa Rica, Cuba, Dominica, the Dominican Republic, Grenada, Guadeloupe, Haiti, Honduras, Jamaica, Martinique, Mexico, Montserrat, Aruba, Curacao, Nicaragua, Panama, El Salvador, Saint Lucia, Saint Vincent and Grenadine, Trinidad and Tobago, American Virgin Islands, British Virgin Islands, Barbados, Antigua and Barbuda, Belize, Bahamas, Bermuda, Puerto Rico, Bonaire St Eustatius and Saba, Saint Martin, Saint-Barthélemy
**South America**	Guatemala, Argentina, Bolivia, Brazil, Guyana, Chile, Columbia, Ecuador, French Guyana, Paraguay, Peru, Surinam, Uruguay, Venezuela
**Oceania/Pacific**	American Samoa, Australia, Salomon Islands, Cook islands, Fiji, French Polynesia, Tonga, Tuvalu, New Zealand, Federated states of Micronesia, Samoa, New Caledonia, Papua New Guinea, Palau, Christmas Island, Kiribati, Vanuatu

Based on the classification previously published in: Rabanal KS, Selmer RM, Igland J, Tell GS, Meyer HE. Ethnic inequalities in acute myocardial infarction and stroke rates in Norway 1994-2009: a nationwide cohort study (CVDNOR). BMC Public Health. 2015;15:1073.

Supplemental Table 2. Age standardized incidence rates of acute myocardial infarction, for men and women from different birth regions for three periods within the study period.

	1999-2005			2006-2012			2013-2019		
Birth region	**N cases**	**Person-years**	**Age-standardized incidence rates (95% CI) per 100 000 PY**	**N cases**	**Person-years**	**Age-standardized incidence rates (95% CI) per 100 000 PY**	**N cases**	**Person-years**	**Age-standardized incidence rates (95% CI) per 100 000 PY**
Men									
Norwegian, n*= 1 561 709	49 416	6 988 948	740 (733-746)	47 338	7 491 215	651 (645-657)	41 865	7 826 772	498 (494-503)
Western Eur, n=69 846	904	160 760	713 (663-762)	1 026	207 982	595 (556-635)	1 144	300 180	450 (422-477)
Eastern Eur, n=77 170	162	18 782	865 (733-998)	195	59 703	623 (517-730)	588	317 566	435 (372-499)
Former Yug, n=14 355	188	37 063	758 (627-890)	314	53 497	744 (649-838)	386	71 761	686 (607-766)
North Africa, 6 416	28	15 580	305 (151-460)	61	22 310	492 (312-672)	82	31 251	394 (292-496)
Sub-Sarahan Africa, n=20 859	41	25 140	243 (121-365)	97	49 790	414 (295-533)	188	95 449	380 (304-457)
Middle Eastern, n=26 203	150	48 041	697 (505-889)	370	88 626	672 (574-771)	522	131 171	601 (527-674)
South Asian, n=18 975	341	51 896	907 (770-1045)	540	70 954	1062 (934-1190)	737	96 199	949 (871-1028)
East Asia, n=3 367	15	7 980	436 (206-666)	23	10 513	340(186-495)	37	14 984	321 (200-443)
Southeast Asia, n=9 191	66	25 817	402 (285-518)	93	36 521	400 (303-496)	149	49 537	411 (333-488)
Central Asia, n=3 305	7	1 297	730 (87-1373)	31	7 644	684 (379-990)	66	16 421	628 (436-819)
North America, n=4 887	90	13 667	556 (438-675)	73	15 226	479 (368-589)	70	18 045	423 (319-527)
Central America, n=1 270	7	1 888	557 (22-1092)	6	3 167	622 (16-1228)	10	5 850	327 (116-539)
South America, n=5 807	42	15 415	592 (270-914)	56	19 936	378 (241-515)	90	28 188	357 (263-451)
Oceania/Pacific, n=1097	7	1 340	1412 (295-2529)	7	2 411	620 (0-1262)	9	4 621	526 (112-939)
Total, n=1 842 457	51 464	7 413 614	740 (734-746)	50 230	8 139 495	651 (645-656)	45 943	9 007 995	500 (495-504)
Women									
Norwegian, n=1 570 396	22538	7 212 809	290 (287-294)	19 603	7 598 462	252 (249-256)	17 346	7 872 988	197 (194-200)
Western Eur, n=56 534	414	172 668	238 (215-261)	422	197 381	213 (193-234)	357	247 459	145 (130-160)
Eastern Eur, n=47 318	39	33 252	242 (165-320)	58	72 647	175 (121-229)	134	21 3702	173 (135-211)

Former Yug, n=13 118	75	33 575	359 (271-446)	127	50 167	380 (310-449)	108	67 344	242 (192-293)
North Africa, n=3 401	-	5 543	103 (0-260)	9	10 297	236 (48-423)	14	17 247	211 (77-345)
Sub saharan Africa, n=17 050	10	15 095	171 (60-281)	19	37 007	176 (86-265)	36	81 209	143 (86-199)
Middle East, n=18 087	28	26 364	292 (165-418)	62	56 619	305 (211-399)	86	94 128	221 (163-279)
South Asia, n=15 802	58	37 682	359 (239-479)	145	58 995	500 (398-603)	195	84 883	377 (313-440)
East Asia, n=5 219	7	10 445	113 (26-199)	10	15 803	139 (51-227)	9	25 117	62 (18-106)
Southeast Asia, n=28 607	25	44 927	207 (107-306)	43	82 688	146 (88-204)	85	150 753	143 (102-184)
Central Asia, n=3 138	-	1 032	242 (0-574)	7	6 786	222 (46-398)	18	15 624	220 (101-339)
North America, n=5 376	69	17 957	259 (195-323)	43	18 289	211 (146-277)	32	19 608	161 (103-219)
Central America, n=1 904	-	2 664	94 (0-240)	5	5 197	260 (0-540)	6	9 759	162 (21-303)
South America, n=7 766	15	15 005	237 (99-374)	21	23 004	166 (79-253)	32	38 732	163 (91-234)
Oceania/Pacific n=674	-	1 599	0 (0-0)	5	1 860	306 (37-576)	-	2 640	135 (0-293)
Total, n=1 794 390	23 284	7 630617	289 (285-292)	20 579	8 235202	251 (248-255)	18 461	8 941 193	194 (192-197)

CI, Confidence Interval; PY, Person-years. *n=number of individuals in the age range 35-79 years who were included in the population at risk at least one year during the study period. Blank cells represent numbers that are not displayed due to less than 5 cases.

Supplemental Table 3. Age standardized incidence rates of stroke, for men and women from different birth regions for three periods within the study period.

	1999-2005			2006-2012			2013-2019		
Men	**N cases**	**Person-years**	**Age-standardized incidence rates (95% CI) per 100 000 PY**	**N cases**	**Person-years**	**Age-standardized incidence rates (95% CI) per 100 000 PY**	**N cases**	**Person-years**	**Age-standardized incidence rates (95% CI) per 100 000 PY**
Norwegian, n*= 1 561 811	28 617	7 021 023	433 (428-439)	27 987	7 532 409	391 (387-396)	26 889	7 866 095	322 (318-326)
Western European, n=69 852	492	161 480	420 (381-460)	595	208 911	366 (334-399)	785	301 146	331 (306-356)
Eastern European, n=77 186	87	18 896	475 (375-575)	90	59 956	322 (241-403)	287	318 249	267 (212-322)
Former Yugoslavian, n=14 361	80	37 288	374 (280-468)	126	53 969	370 (298-443)	140	72 383	285 (230-341)
North African, n=6 417	11	15 625	215 (50-380)	27	22 389	286 (142-431)	35	31 388	189 (109-268)
Sub-Sarahan African, n=20 849	27	25 147	190 (65-315)	78	49 830	277 (188-365)	156	95 489	301 (233-369)
Middle Eastern, n=26 221	58	48 245	371 (198-544)	101	89 287	275 (197-353)	183	132 244	244 (195-292)
South Asian, n=18 982	112	52 453	444 (315-572)	148	72 011	410 (313-507)	237	97 679	375 (319-432)
East Asian, n=3 367	21	7 960	492 (271-713)	19	10 514	255 (125-385)	38	14 993	386 (238-534)
Southeast Asian, n=9 192	61	25 807	512 (359-666)	73	36 582	373 (273-473)	118	49 581	348 (277-420)
Central Asian, n=3 305	-	1 316	0	7	7 695	246 (0-512)	20	16 538	283 (115-451)
North American, n=4 890	43	13 728	245 (170-319)	59	15 275	380 (282-478)	33	18 115	214 (136-291)
Central American, n=1 268	-	1 900	397 (0-901)	9	3 160	575 (104-1046)	7	5 849	280 (66-494)
South American, n=5 806	13	15 475	129 (51-206)	28	20 021	262 (124-401)	32	28 347	174 (92-255)
Oceania/Pacific, n=1 097	-	1 346	566 (0-1355)	7	2 421	723 (0-1481)	7	4 624	396 (11-781)
Total, n=1 824 604	29 628	7 447 689	432 (427-437)	29 354	8 184 430	389 (385-394)	28 967	9 052 720	320 (316-324)
Women									
Norwegian, n=1 569 797	21 854	7 206 310	283 (279-286)	19 182	7 595 297	248 (245-252)	17 699	7 872 688	203 (200-206
Western European, n=56 515	439	172 431	254 (231-278)	430	197 263	222 (200-243)	415	247 362	170 (154-187)
Eastern European, n=47 313	42	33 216	221 (151-292)	74	72 568	202 (146-257)	165	213 635	195 (156-235)

Former Yugoslavian, n=13 119	75	33 532	385 (292-477)	94	50 227	291 (229-353)	89	67 428	206 (159-253)
North African, n=3 400	5	5 532	367 (0-751)	11	10 291	195 (61-330)	20	17 249	252 (115-389)
Sub-Sarahan African, n=17 042	13	15 092	163 (52-273)	24	36 966	186 (91-281)	82	81 122	264 (189-339)
Middle Eastern, n=18 082	24	26 345	227 (109-345)	54	56 626	254 (172-337)	84	94 165	189 (138-241)
South Asian, n=15 802	53	37 683	288 (182-395)	88	59 066	305 (225-386)	125	85 122	234 (184-284)
East Asian, n=5 218	22	10 423	378 (214-542)	20	15 758	208 (110-306)	18	25 065	92 (44-140)
Southeast Asian, n=28 603	61	44 804	322 (217-428)	87	82 590	231 (163-299)	172	150 550	237 (187-286)
Central Asian, n=3 136	-	1 031	189 (0-555)	7	6 787	272 (0-553)	19	15 632	288 (142-434)
North American, n=5 372	60	17 930	228 (167-289)	63	18 255	291 (217-365)	30	19 604	152 (97-208)
Central American, n=1 903	-	2 659	237 (0-483)	-	5 199	143 (0-301)	9	9 746	176 (42-309)
South American, n=7 766	15	14 991	212 (82-343)	20	22 998	122 (57-187)	41	38 709	169 (106-233)
Oceania/Pacific, n=673	-	1 597	128 (0-308)	-	1 858	123 (0-293)	-	2 639	143 (0-309)
Total, n=1 793 741	22 670	7 623 576	282 (278-286)	20 160	8 231 749	247 (244-251)	18 971	8 940 716	202 (199-205)

CI, confidence interval; PY, Person-years. *n=number of individuals in the age range 35-79 years who were included in the population at risk at least one year during the study period. Blank cells represent numbers that are not displayed due to less than 5 cases.

Supplemental Table 4. Average annual percent change in acute myocardial infarction incidence rates in individuals aged 35-79 years in Norway during 1996-2019.

	**Region of birth**	**Average annual change (IRR -1)** [a] **(95% CI)**	**P-value**	**P for interaction**[b]
**Men**				
	Norway	-2.4% (-3.0%, -1.9%)	<0.001	Ref
	Western Europe	-3.1% (-3.8%, -2,4%)	<0.001	0.165
	Eastern Europe	-3.6% (-4.7%, -2.5%)	<0.001	0.004
	Former Yugoslavia	-0.9% (-2.1%, 0.4%)	0.190	0.043
	South Asia	-1.4% (-2.5%, -0.4%)	0.009	0.272
**Women**				
	Norway	-2.0% (-2.4%, -1.7%)	<0.001	Ref
	Western Europe	-3.1% (-4.0%, -2.2%)	<0.001	0.064
	Eastern Europe	-1.4% (-3.6%, -0.9%)	0.233	0.848
	Former Yugoslavia	-2.3% (-4.1%, -0.4%)	0.018	0.838
	South Asia	-0.7% (-2.5%, 1.0%)	0.421	0.104

IRR, Incidence rate ratio. [a]The average annual changes are based on incidence rate ratios from Poisson regression or negative binomial regression (nbreg was used when goodness of fit tests after Poisson regression was significant), adjusted for age. [b]P for interaction refers to a test of interaction between calendar year and birth region with Norwegian-born as reference group in a separate Poisson/negative binomial regression model.

Supplemental Table 5. Average annual percent change in total stroke incidence rates in individuals aged 35-79 years in Norway during 1996-2019.

	**Region of birth**	**Average annual change (IRR -1)** [a] **(95% CI)**	**P-value**	**P for interaction**[b]
Men				
	Norway	-1.6% (-1.8%, -1.3%)	<0.001	Ref
	Western Europe	-1.6% (-2.4%, -0.9%)	<0.001	0.946
	Eastern Europe	-3.4% (-5.0%, -1.8%)	<0.001	0.001
	South Asia	-2.1% (-3.6%, -0.6%)	0.006	0.538
	Former Yugoslavia	-2.4% (-4.2%, -0.7%)	0.007	0.344
Women				
	Norway	-1.9% (-2.2% -1.7%)	<0.001	Ref
	Western Europe	-2.8% (-3.6% -1.9%)	<0.001	0.085
	Eastern Europe	-1.8% (-3.8%, 0.2%)	0.082	0.967
	South Asia	-2.7% (-4.7%, -0.6%)	0.012	0.465
	Former Yugoslavia	-4.3% (-6.3%, -2.3%)	<0.001	0.020

IRR, Incidence rate ratio. [a]The average annual changes are based on incidence rate ratios from Poisson regression or negative binomial regression (nbreg was used when goodness of fit tests after Poisson regression was significant), adjusted for age. [b]P for interaction refers to a test of interaction between calendar year and birth region with Norwegian-born as reference group in a separate Poisson/negative binomial regression model.
